# Denervation of the Patella During Knee Arthroplasty: An Updated Systematic Global Review

**DOI:** 10.3390/jcm13226942

**Published:** 2024-11-18

**Authors:** Kennedy Nkachukwu, Amanda Alejo, Jeffrey Toman, Jalal Jwayyed, Justin Iwuagwu, Andrew Alejo

**Affiliations:** 1 College of Medicine, Northeast Ohio Medical University, Rootstown, OH 44272, USA; 2 Department of Orthopaedics, Stony Brook University Hospital, Stony Brook, NY 11794, USA

**Keywords:** total knee arthroplasty, osteoarthritis, patella denervation, anterior knee pain

## Abstract

**Background:** Total knee arthroplasty is a widely endorsed surgical intervention, extensively recognized within the orthopedic field for its efficacy in significantly reducing pain and enhancing overall mobility in patients suffering from advanced stages of osteoarthritis. Despite a general consensus on the core procedural steps, the rapid advancements in implant technology and the nuanced techniques in knee reconstruction have inevitably introduced subtle variations in surgical approaches. These variations often emerge due to individual surgeon preferences, their unique expertise, and comfort levels with specific techniques. Anterior knee pain, however, remains a frequent postoperative complication, likely attributed to the extensive and complex innervation of the patella. To address this challenge, some surgeons have adopted patellar denervation, hypothesizing that by reducing nerve signaling from the patellar region, patients may experience a measurable decrease in pain. **Methods:** A systematic search was performed to include eight recent level I studies to analyze this issue. **Results:** Of the eight reviews, there were four strong studies that concluded patellar denervation helps decrease anterior knee pain in the acute period, but this may not last long term. The other four papers did not show a difference in anterior knee pain after denervation. **Conclusions:** This review synthesizes and critically analyzes the current body of literature, aiming to provide clinicians with evidence-based insights into the potential benefits and limitations of incorporating patellar denervation into their surgical especially during the acute post-operative period.

## 1. Introduction

Total knee arthroplasty (TKA) is widely recognized for its ability to alleviate pain and enhance functional outcomes in patients with primary osteoarthritis [1]. It is well known that the pathology of osteoarthritis involves inflammatory cytokines, such as MMP-13, MMP-1, IL-1, and IL-6, that lead to collagen breakdown which impedes joint function [2]. Although the clinical presentation can differ among patients, with a spectrum of symptoms exhibited, the most common complaints from patients include pain at the joint, swelling, stiffness, erythema, and difficulty ambulating [3]. Various factors have been associated with knee arthritis which include female sex, obesity, older age, heavy lifting or squatting, and malrotation of the knee [4].

Although extensive research has been completed to continue improvement of osteoarthritis treatments, advancements in technology have introduced variations in the procedure, meaning that not all knee replacements follow the same methodology. A conventional approach to knee arthroplasty provides sufficient visualization for precise implant placement and allows ample space for robotic assistance, aiding surgeons in achieving accurate bone cuts. While the fundamental steps of knee arthroplasty remain consistent, individual surgeons may incorporate specific modifications based on their preferences. Specifically, denervation of the patella (PD) involves electrocautery that is used circumferentially to cause a disruption to the nerves that innervate the patella [5].

Despite improvements in surgical techniques, many patients continue to experience anterior knee pain (AKP) after knee arthroplasty, which can significantly impair quality of life [6,7,8]. In response, orthopedic surgeons have conducted numerous studies to assess whether additional procedural modifications can reduce postoperative anterior knee pain. Denervation of the patella, aimed at reducing sensory nerve input to this area, is believed to help alleviate such pain. Complete circumferential electrocautery is the most commonly used technique to successfully sever the nerve endings [9,10,11]. This is utilized due to the vast nerve supply that the patella is innervated by. These nerves include the anterior femoral cutaneous nerve, medial cutaneous nerve of the thigh, lateral femoral cutaneous nerve, and medial and lateral retinacular nerves [12,13]. By interrupting these nerve pathways during knee arthroplasty, some surgeons hope to mitigate postoperative AKP.

This review aims to compile and present the latest research findings on patellar denervation in knee arthroplasty, providing orthopedic surgeons with an up-to-date resource to guide their clinical decision-making. We hope this synthesis will support surgeons in making informed choices regarding patellar denervation as a potential strategy for enhancing patient outcomes in knee arthroplasty.

## 2. Anatomy and Indications for Knee Arthroplasty

The knee is a synovial hinge joint that connects the femur, tibia, and patella, essential for supporting body weight, maintaining stability, and enabling leg movement [14]. Primarily, it facilitates flexion, extension, and limited rotation. The medial and lateral femoral condyles engage with the tibial plateau, while the distal femur articulates with the patella to aid knee extension. Key muscle groups, the quadriceps and hamstrings, are integral to knee extension and flexion, respectively, thus contributing significantly to the joint’s stability and functionality. The knee’s structural integrity is fortified by four principal ligaments: the medial and lateral collateral ligaments, which restrict excessive medial and lateral movement, and the anterior and posterior cruciate ligaments, which prevent anterior and posterior displacement of the tibia. Together, these ligaments play a crucial role in maintaining the joint’s alignment and preventing dislocation.

The knee joint is equipped with two types of cartilage, each essential for smooth, low-friction articulation. Articular cartilage, which lines the joint surfaces, enables bones to glide past each other seamlessly [15]. Meanwhile, fibrocartilage, composed of dense fibrous material, forms the menisci of the knee. These C-shaped structures, the medial and lateral menisci, lie between the femur and tibia, providing stability and absorbing axial forces [16]. Supporting the knee’s complex movements are four primary bursae, small sacs filled with synovial fluid that significantly reduce friction. These bursae include the suprapatellar, located between the quadriceps femoris and femur; the prepatellar, located between the patella’s apex and the skin; the infrapatellar, situated between the tibia and patellar ligament; and the semimembranosus, positioned between the semimembranosus muscle and the medial head of the gastrocnemius [17]. Together, these bursae and cartilage structures support the knee’s stability, alignment, and efficiency in movement.

The knee joint is particularly prone to osteoarthritis due to several critical factors. As the largest joint in the body, it bears substantial body weight, subjecting it to considerable strain through everyday activities such as standing and running [18]. Over time, this persistent stress contributes to cartilage degradation [19]. Additionally, factors like high body mass index, aging, previous knee trauma, athletic activity, and genetic predisposition further elevate the risk of developing osteoarthritis [20]. Notably, increased body weight accelerates wear on the knee joint, a condition notably prevalent in the United States. As the knee cartilage erodes, direct bone-on-bone contact ensues, exacerbating joint damage and triggering inflammation. Without the cushioning effect of cartilage, underlying bone nerves become exposed, resulting in pain and discomfort, especially during activities like bending, running, or standing.

Various treatments exist for osteoarthritis, each offering differing degrees of effectiveness. Weight loss serves as a highly beneficial and sustainable approach, as it alleviates stress on the joint [21]. Among pharmaceutical options, over-the-counter pain relievers, particularly non-steroidal anti-inflammatory drugs (NSAIDs), are commonly prescribed due to their efficacy in reducing osteoarthritis pain; however, prolonged use of NSAIDs may heighten risks for cardiovascular events, including heart attack, stroke, and heart failure [22,23]. Corticosteroid injections also provide significant pain relief and enhance knee function, though they are not advisable for extended use [24,25]. For advanced cases, one of the most effective interventions is total knee arthroplasty. This procedure replaces damaged joint tissue with metal and polyethylene components, facilitating smoother knee movement and eliminating bone-on-bone friction and associated pain. While TKA is highly effective in alleviating osteoarthritis symptoms, it can sometimes affect small nerves, potentially leading to chronic knee discomfort after surgery [26].

Total knee arthroplasty is performed when the patient has osteoarthritis of both compartments. If there is arthritis of just one compartment, usually the medial side due to the wear pattern and weight distribution biomechanics, a unicompartmental knee replacement can be explored [27,28]. Various techniques and different implants have been shown to be effective in total knee arthroplasty, but the main question is usually to press-fit or cement the implants into the bone. Recently, it has been shown that both options provide successful outcomes, so this is another factor that is up to the surgeon’s discretion [2].

**Figure 1:**
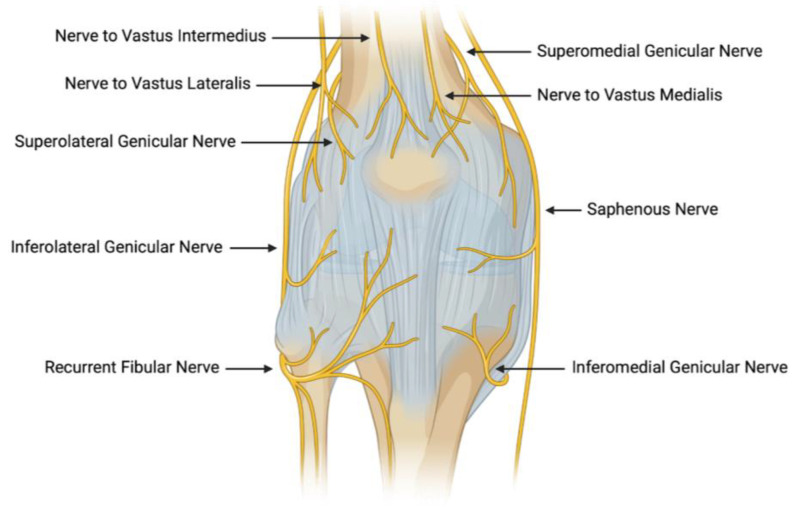
Innervation supply to the knee.

The knee joint is extensively innervated, a characteristic that contributes significantly to its susceptibility to pain. Motor innervation is provided by several key nerves, including the femoral, tibial, and peroneal nerves [29]. The femoral nerve, the largest of the lumbar plexus, controls the anterior knee and enables knee extension. The tibial nerve governs motor function in the posterior knee, facilitating knee flexion, while the peroneal nerve innervates the anterior and lateral compartments, enhancing joint mobility and stability. Primary sensory innervation is supplied by the infrapatellar branch of the saphenous nerve, responsible for sensation in the anterior knee, and the genicular nerves, which innervate the joint capsule and surrounding tissues [30]. Specifically, the patella is innervated by the lateral and medial superior genicular nerves, which are principal contributors to postoperative knee pain [12] (Figure 1). Consequently, these genicular nerves are often targeted and ligated during patellar denervation procedures to mitigate post-surgical knee pain. A thorough understanding of these structures and their sensory innervation is essential in knee surgeries where nerve denervation may be performed to reduce postoperative anterior knee pain.

## 3. Surgical Technique

Total knee arthroplasty is a well-established surgical intervention for relieving pain and enhancing function in patients with advanced osteoarthritis of the knee. Among various surgical techniques, the medial parapatellar approach is widely favored due to its adaptability, superior joint exposure, and procedural efficiency [31]. In cases where the knee exhibits increased valgus or varus alignment, additional corrective steps are implemented to realign these deformities and achieve optimal knee stability and kinematics.

The patient is positioned supinely at the edge of the operating table to allow for full manipulation of the knee. Key anatomical landmarks, including the tibial tubercle, patella, and patellar ligament, are identified to initiate the procedure. An incision is made along the medial border of the patella, which starts proximally by the quadriceps tendon and is extended distally toward the tibial tubercle. This approach facilitates efficient patellar eversion and provides optimal visualization of the knee joint, supporting precise bone preparation and balanced soft tissue handling. Caution is taken to preserve extra soft tissue for closure. Additionally, a portion of the medial capsular sleeve is removed to expose the knee joint fully.

The knee is then flexed to 90 degrees, and the patella is everted. The fat pad, residual anterior cruciate ligament, and lateral meniscal horn are resected to enhance access. To establish correct femoral rotation, a femoral intramedullary guide is inserted, followed by secure placement of a femoral cutting jig on the distal femur. Using this guide, a precise posterior femoral cut is executed. The appropriate implant size is assessed by positioning a guide on the cut femur, which is then replaced by an anterior femoral cutting block to perform an anterior cut on the distal femur. Attention then shifts to the tibia, where an extramedullary tibial guide is aligned with the bone. The knee’s alignment and flexion are meticulously verified before making the tibial cut. Upon completion, the tibial guide, along with excess meniscal tissue and posterior osteophytes, is carefully removed to ensure an optimal fit and alignment for the implant.

A trial tibial base plate is selected for appropriate sizing and rotational alignment. A femoral trial component is then positioned, followed by knee extension and patellar eversion. At this stage, the surgeon decides on patellar resurfacing or denervation. If denervation is chosen, electrocautery of the lateral genicular nerve is performed, due to its innervation of the patella (Figure 1). A patellar button is carefully sized and aligned with the femoral component. The knee is then assessed for stability across various positions, including varus and valgus forces, posterior dropping, and tightness or looseness in flexion and extension. After confirming fit, the trial components are removed, and the permanent implants are either cemented or press-fitted into place. If cemented, any excess material is meticulously cleared. The knee undergoes a final examination to confirm full range of motion before closure is completed [32].

Less commonly utilized approaches, such as the subvastus, midvastus, and lateral parapatellar techniques, are designed to preserve the quadriceps mechanism or minimize the necessity for lateral releases in cases of valgus deformity [33,34]. Despite these potential advantages, these approaches present greater technical complexity and are, therefore, less frequently adopted [35]. Consequently, the medial parapatellar approach remains the gold standard, favored for its straightforward execution, reproducibility, and consistently reliable outcomes in pain relief, functional recovery, and prosthesis longevity.

## 4. Materials and Methods

This systematic review was conducted following the JBI methodology and PRISMA guidelines for systematic reviews

### 4.1. Review Question

Our research question was the following: “Does circumferential patellar denervation after total knee arthroplasty reliably reduce postoperative anterior knee pain compared to non-denervation?”.

### 4.2. Eligibility Criteria

The clinical studies qualified for inclusion if they met the following population, concept, and context (PCC) criteria. Population (P): The population criteria permitted studies with participants regardless of age or gender that have undergone total knee arthroplasty. Concept (C): The included studies must evaluate and compare the outcomes of patellar denervation vs. non-denervation with a specific focus on pain in the short and long term as the primary outcome utilizing clinical scores such as the visual analogue scale (VAS), American Knee Society score (KSS), Kujala score, Oxford knee score (OKS), Bartlett patellar score, and Western Ontario and McMaster Universities Osteoarthritis Index (WOMAC). Context (C): The studies had to be randomized controlled trials (RCTs) with a follow-up period of at least 6 months. Additionally, the context included settings such as clinics, hospitals, or rehabilitation centers for the knee surgeries and postoperative evaluation. Only human studies published in English from January 2014 to October 2024 were included.

### 4.3. Exclusion Criteria

Studies that did not meet the specific PCC criteria were excluded.

### 4.4. Search Strategy

A comprehensive search strategy was employed to find relevant studies for inclusion. This strategy was employed across three databases to ensure an adequate thorough examination of the literature. We searched PubMed, Cochrane Library (CENTRAL), and MEDLINE with a combination of MeSH terms and keywords from 2 October to 15 October 2024, limiting the search to papers published between January 2014 and October 2024.

### 4.5. Study Selection Process

After filtering for human clinical trials published from 2014 to 2024 written in English, 35 articles were identified through this search method. After removing duplicates, 21 articles remained and were subsequently screened by reading the titles and abstracts. Of these 21, 12 articles were excluded for not meeting the inclusion criteria. The full text of the final eight articles were assessed and included in this systematic review. No additional studies were identified through hand-searching reference lists or contacting authors as full-text access was available.

The assessment of inclusion for the selected studies involved two processes. First, we screened study titles and reviewed the abstracts. Then, we assessed the full text. This process was conducted on an individual basis by two authors, with any disagreements being resolved by a third. The process adheres to the PRISMA 2020 guidelines for transparency and reliability.

### 4.6. Data Extraction and Data Synthesis

**Figure 2:**
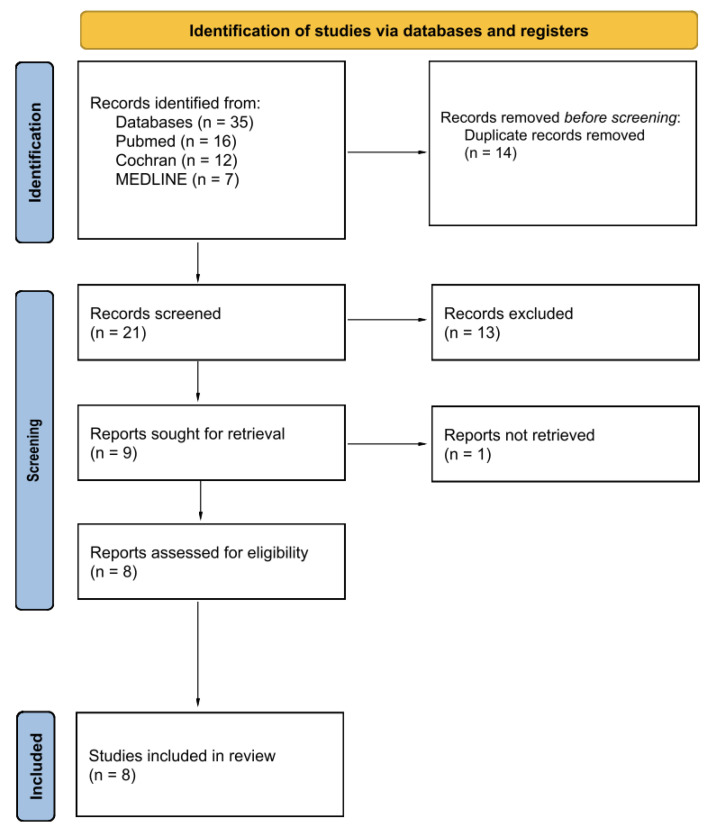
Search model for publications.

This review’s data were extracted using a form based on the JBI tool to encapsulate key details such as authorship, publication year, study design, patient characteristics, outcomes, interventions, and other relevant data. Subsequently, descriptive analyses of these data were conducted (Figure 2). 

## 5. Outcomes and Results

A total of eight studies were included in this review (Table 1). Six of the eight studies were randomized control studies, with the other two being prospective studies. A total of 1244 patients were included in these studies. Total patients, sex, mean age, mean follow-up, and the minimum and maximum follow-up were evaluated (Table 1). Additionally, the level of evidence, summarized results, benefit in AKP, strengths and weaknesses, and JBI score were also evaluated (Table 2).

The findings regarding the effectiveness of patellar denervation in reducing AKP after knee arthroplasty are varied. Several studies show significant benefits, particularly in the short term. For instance, Alomran demonstrated that patients who underwent patellar denervation after non-resurfaced total knee arthroplasty experienced significantly less AKP, with only 9% reporting pain compared to 33% in the non-denervation group after 24 months (*p* = 0.02) [36]. Similarly, Thiengwittayaporn et al. found that patellar denervation led to a lower incidence of AKP in patellar resurfacing total knee arthroplasty patients (6.4% vs. 16.2%, *p* = 0.032) and reduced AKP intensity at three months (17.1 ± 8.0 vs. 54.0 ± 14, *p* = 0.017), though these differences did not persist at later time points [40]. Sun et al. also observed that patellar denervation reduced AKP in unicompartmental knee arthroplasty, though specific AKP scores were not provided [39].

However, other studies present more mixed results, indicating that the benefits of patellar denervation may not be sustained over time. Van Jonbergen et al. initially reported promising results, with a lower prevalence of AKP in the intervention group (26%, 95% CI 18 to 35) compared to the control group (38%, 95% CI 29 to 48) at one year, but the difference was not statistically significant (*p* = 0.06), and the benefits did not last beyond 3.7 years [41]. Similarly, Pulavarti et al. found significant short-term improvements in AKP at three months after surgery (*p* < 0.05), but these benefits were not sustained at the 12- and 24-month follow-up points [38].

On the other hand, some studies found no significant benefit from patellar denervation at all. Spencer et al. reported no clinically relevant improvement in AKP after patella rim electrocautery in patients undergoing total knee arthroplasty without patella resurfacing [11]. Budhiparama et al. found no difference in AKP between patients who underwent circumferential patellar cauterization and those who did not, with mean visual analog scale (VAS) scores being nearly identical between groups (3 ± 0.9 vs. 3 ± 0.7, *p* = 0.920) at two years [10]. Lastly, Goicoechea et al. concluded that patellar denervation did not improve AKP in primary total knee arthroplasty with patellar resurfacing, showing mild differences in VAS score for stairs in favor of the non-denervation group (2.9 vs. 1.5, *p* = 0.003) at one year [37].

## 6. Discussion

Total knee arthroplasty serves as an effective intervention for osteoarthritis, allowing for reduced pain and improved mobility. Postoperative pain may act as a barrier to proper rehabilitation, impacting quality of life and mobility. The question of whether to denervate the patella is crucial to consider, as it can prevent pain during the recovery period, which may lead to better clinical outcomes. In this review, 1244 total patients underwent a knee arthroplasty. The outcomes between studies primarily focused on incidence of pain, joint motion, and function.

Strong clinical evidence suggests that denervation of the patella leads to pain reduction, increased range of motion, and better overall function. In a study by Alomran, 184 patients were evaluated who were randomly and equally assigned to the denervation and control groups [36]. Circumpatellar electrocautery was performed on the denervation group, and both groups had the same postoperative management. The effect of denervation was assessed via AKP ratings, WOMAC, and range of motion at a follow-up interval of six weeks, three months, and annually. Overall, this study showed promising results at various follow-up periods. They found that the average WOMAC score was significantly lower at the one-year period for the denervation group (17.1 versus 21.6). This supports the use of denervation in TKA as lower scores demonstrate an improvement in pain, stiffness, and overall function. Additionally, 9% of the denervation group compared to 33% of the non-denervation group reported AKP at the 24-month follow-up period. Pain reduction was therefore present in the acute postoperative period and able to be maintained in a more long-term fashion, establishing the benefit of PD. Lastly, the average range of motion was higher in the denervated patients, at 126.7° ± 8.3, compared to 112.5° ± 4.8 in the control group. It is possible that with lowered postoperative pain, patients have more tolerability of their rehabilitation, which may contribute to achieving a better range of motion compared to those who experience more discomfort.

While PD has primarily been studied in TKA, there is also research into the impact of PD in unicompartmental knee arthroplasty (UKA). Sun et al. studied PD in UKA in 120 patients, assessing pain, joint function, and kneeling ability through Kujala, HSS, VAS, and FJS-12 scores at one, six, and twelve months [39]. They found that there was a statistically significant difference in Kujala scores at both the 6-month and 12-month period. This indicates that there was overall better patellar function in the PD group. They also found that VAS scores were significantly lower in the treatment group at both time periods, while FJS-12 scores were significantly higher. With lower VAS scores showing less pain and higher FJS-12 scores showing an increased degree of “forgetting” about their knee joint, it can be concluded that the alleviation of postoperative pain allows for patients to be less aware of their joint having an impact on their daily activities. This is beneficial in the sense that because of PD, pain is no longer the primary focus of patients during their recovery period, and they can return to their typical routine and degree of movement, achieving better integration of their UKA in their lifestyle. Lastly, the PD group obtained a better actual kneeling ability compared to the non-PD group. Again, this may be related to the improved FJS-12 scores, as patients in the PD group are able to kneel with less trouble, leading to a decreased awareness of their UKA and overall impact on their daily life.

Thiengwittayaporn et al. studied the impact of patellar denervation on anterior knee pain and patient satisfaction after TKA with patellar resurfacing [40]. In total, 228 patients participated in this study, and these outcomes were assessed at various follow-up periods ranging from 3 to 24 months postoperatively. It was found that there was both a significant reduction in the incidence of anterior knee pain in the treatment group, as well as a lower intensity of pain compared to the control group. However, it is important to note that the differences in intensity of AKP were not found to be significant at follow-up points after 3 months. Similarly, while patient satisfaction was significantly higher in the treatment group, this difference was not maintained at later time periods. This study supports the use of PD as it reduces pain and increases patient satisfaction while highlighting an issue of longevity in terms of this effect. Therefore, while there is evidence that confirms the benefits of PD, there may be limitations in the length of time that these benefits last. Overall, these studies provide evidence of the benefits associated with utilization of PD in knee arthroplasty. Across these level I studies, there is a clear connection between PD and a diminished level of anterior knee pain. There is, however, some discrepancy associated with the length of time that this lasts. By implementing PD, the combined result of pain relief and improved joint function can lead to an increased patient satisfaction and “joint forgetting”, which is the ideal clinical outcome for TKA patients, as postoperative pain can have a substantial impact on patients’ quality of life.

Other studies offer mixed results regarding the benefits of PD. Pulavarti et al. studied the impact of circumpatellar denervation in TKA on 126 patients, assessing patient satisfaction, flexion range, and VAS pain scores, in addition to Oxford score, American Knee Society score, American Knee Society function score, Bartlett patella score, activities of daily living score, and UCLA activity scale [38] They found that there was significantly less anterior knee pain in the treatment group at the 3-month follow-up; however, this was not maintained at the 12- and 24-month follow-up. This was evident through a reduction in VAS score as well as through the AKP component of the patellar score. This result is similar to that of Thiengwittayaporn et al., who also specifically highlighted 3 months as the limit for which the pain relief lasts. This may suggest that PD offers pain reduction that is more acute than long-term. Additionally, at 3 months, they detected a significant difference in the patella score. They also found that the degree of flexion present in the treatment group was 103.6°, versus 99.2° in the control group, which was significant. Therefore, although the reduction of pain did not necessarily continue, there was still a clinical benefit in terms of the participants’ recovery and ability to achieve a higher degree of flexion at a later time. As discussed in the study conducted by Alomran, these participants also achieved a better range of motion from PD. Both results suggest that reduction of pain, regardless of duration, seems to positively impact knee function. Early pain control may play an influential role in increasing mobility early in the recovery process, which may lead to better long-term clinical outcomes such as degree of flexion. Lastly, at the 24-month follow-up, patient satisfaction was significantly higher as well. It is interesting to note that even though PD patients were not experiencing less pain at this point, they were still more satisfied than the control group, potentially due to their increased mobility. Overall, this study supports the use of PD in terms of increasing patient satisfaction with TKA, improving flexion, and reducing pain in a short-term setting.

While there is little consensus regarding the duration of pain relief, a study conducted by van Jonbergen et al. specifically focused on this point [41]. They assessed 202 patients at the 3–4-year time point, via the clinical anterior knee pain rating system, WOMAC, and American Knee Society knee and function scores. In one of their previous studies, they found that PD led to a lower prevalence of AKP as well as improved patient-reported outcome measures (PROMs) at the one-year point. However, in the study mentioned previously, they found that although at the 3.7-year mark, prevalence of AKP was 26% in the treatment group and 38% in the control group, this was not statistically significant (*p* = 0.06) and the overall incidence of AKP was unchanged. Additionally, both groups had similar WOMAC and American Knee Society knee and function scores. In addition to Pulavarti et al. and Thiengwittayaporn et al., van Jonbergen et al. also provide evidence that pain relief associated with PD is not maintained in a long-term fashion [38,40,41]. However, they still recommend PD due to the positive results from their previous study and believe that this intervention does reduce pain, just in an acute manner. Between these three studies, there are still questions regarding at what point this reduction in pain is no longer present. However, all provide statistically significant evidence that PD reduces pain postoperatively. Further research should be conducted in regard to this, as it may provide better insight into the extent to which PD can improve clinical outcomes in TKA.

A shared theme in the studies conducted by Budhiparama et al., Goicoechea et al., and Spencer et al. was that PD showed no significant difference in reducing AKP following TKA compared with the control groups [10,11,37]. While each study concluded that denervation did not lead to major improvements in pain reduction or functional outcomes, they varied substantially in terms of study design and execution. Differences included the sample size, the scoring systems used, the surgical techniques, the prosthetics, and patient follow-up duration. Budhiparama et al. was the only study to perform bilateral TKA, where only the right knee was denervated, while the left knee acted as a control, and neither knee underwent patellar resurfacing [10]. This design is significant because most individuals are right-limb dominant, which may have influenced the results. However, the gender ratio was significantly skewed, with 67 women and only 6 men. Budhiparama et al. followed patients at one month, six months, one year, and two years to capture both short- and long-term outcomes [10]. In contrast, Goicoechea et al. conducted a double-blind, randomized controlled trial with unilateral TKA and patellar resurfacing, using three different prosthetic models and varying surgical approaches (posterior-stabilizing vs. cruciate-retaining) based on the surgeon’s criteria [37]. Furthermore, the authors acknowledged that resurfacing the control group’s patellae might have resulted in accidental partial denervation, calling into question the validity of the results. Women were overrepresented in their study as well, with 119 women and 50 men. Patients were only followed up with at one year, which did not allow the opportunity to observe whether there were short-term benefits for patients receiving denervation, which is seen in many studies. Lastly, Spencer et al.’s study was also a double-blind, randomized controlled trial design, focusing on unilateral TKA without patellar resurfacing, with the same implant used across all participants [11]. Their study utilized two surgeons who performed the procedures, which could be a confounder. Furthermore, the randomization was performed for the full sample size rather than in blocks, which led to skewed group sizes, with 93 patients in the control group (no denervation) and 49 patients receiving denervation. Follow-up was carried out at six weeks, one year (the primary outcome), and two years. By the 1-year follow-up, only 81 patients from the control group and 43 patients from the denervation group remained for analysis.

Although these studies concluded that denervation did not significantly improve outcomes, they each had different flaws. The studies varied in terms of surgical approaches, scoring systems, prosthetics, patient populations, and healthcare settings. The United States healthcare system and patient population vary greatly compared to Indonesia, Spain, and the United Kingdom. Budhiparama et al. showed no improvement in AKP with denervation in a bilateral TKA, and the fact that only the right knee was denervated in a right-limb dominant population may have influenced the results [10]. Goicoechea et al. similarly found no added benefit in unilateral TKA with patellar resurfacing, but the use of three different prosthetic models and varied surgical approaches could have introduced confounding variables [37]. The authors also noted that accidental partial denervation in the control group through resurfacing may have affected the results. Finally, Spencer et al. demonstrated similar findings in a unilateral TKA without resurfacing, despite the skewed randomization and the fact that fewer patients completed follow-up in the denervated group [11]. The common theme across these studies is the conclusion that there is a lack of significant clinical benefit with denervation in bilateral or unilateral TKA in both resurfaced and non-resurfaced knees.

When evaluating the limitations of our systematic review, the included studies were conducted in various regions like Indonesia, Spain, the United Kingdom, the Netherlands, Saudi Arabia, China, and Thailand. These populations differ greatly in terms of demographics, healthcare systems, and cultural factors. This may make it hard to apply the findings to a U.S. population or country-specific population. Likewise, since this review included only eight studies and a total of 1244 patients, this analysis might not provide enough data to confidently say whether patellar denervation works well in the long term due to the limited search. Furthermore, some studies did not measure short-term results, leaving gaps in understanding whether denervation might be valuable for immediate pain relief, even if long-term benefits are uncertain. As with systematic reviews, there is always a degree of publication bias where the publications included showed promising results in regard to the hypothesis.

## 7. Conclusions

Based on evidence from multiple level I studies, patellar denervation in TKA provides significant short-term AKP relief, improving patient satisfaction without added complications. It is a quick intervention that does not add much operative time and is simple to complete using electrocautery. While there is still uncertainty relating to the duration of time this relief lasts, there is consensus among these studies that it will reduce pain in the acute period and allow for the achievement of increased mobility in terms of range of motion, flexion, and kneeling ability. These factors all contribute to patient satisfaction. As a result of these findings, patellar denervation can be used as an intervention for reducing postoperative pain in an acute setting. Further studies should be conducted to evaluate for additional data collection in both the acute and long-term time periods. This will allow for more temporal data that can aid in the support of denervating the patella.

## Figures and Tables

**Table 1:** Summary of publication characteristics.

Study	Study Type	Total Patients	Sex (F/M)	Mean Age	Mean Follow-Up (Months)	Minimum Follow-Up (Months)	Maximum Follow-Up (Months)
Alomran (2015) [36]	Randomized, controlled trial	184	Not reported	Not reported	37.4 − 39.0	24	1
Budhiparama et al. (2019) [10]	Prospective study/quasi-randomized	73	67/6	66 years	30 ± 5.9	24	45
Goicoechea et al. (2020) [37]	Prospective, randomized, double-blind trial	169	119/50	72.7 years	12	12	12
Pulavarti et al. (2014) [38]	Randomized, controlled trial	126	68/58	69.9 Years	26.3–26.5	24	26.5
Spencer et al. (2023) [11]	Randomized, controlled trial	142	74/68	71 years	24	24	24
Sun et al. (2024) [39]	Randomized, controlled trial	120	79/41	Not reported	Not Reported	6	12
Thiengwittayaporn et al. (2020) [40]	Randomized, controlled trial	228	175/53	Not reported	24	24	24
Van Jonbergen et al. (2014) [41]	Randomized, controlled trial	202	140/62	70.5 Years	44.4	13.2	50.4

**Table 2:** Analysis of publication data results.

Study	Study Type	Level of Evidence	Total Patients	Summarized Results	Benefit in AKP	Strengths and Weaknesses	JBI Score
Alomran (2015) [36]	Randomized, controlled trial	RCT/level I	184	At 24 months post operation, 9% in the denervation group and 33% in the non-denervation group reported anterior knee pain (grade I–III). This difference was statistically significant (*p* = 0.02). Mean postop WOMAC score for the denervation group at one year was 17.1 (3–25.4), while for the non-denervation group, it was 21.6 (0–32.8) (*p* = 0.03). Mean postop range of motion was 126.7 ± 8.3° for the denervation group and 112.5 ± 4.8° for the non-denervation group, (*p* = 0.02).	Benefit	Strengths: Randomized, double-blinded, well-matched groups, validated outcome measures, appropriate statistical analysis. Weaknesses: Unclear allocation concealment, no surgeon blinding, no intention-to-treat analysis.	10/13
Budhiparama et al. (2019) [10]	Prospective study	Prospective study/level IV	73	No differences were found in the mean VAS score between the cauterized and non-cauterized knees (3 ± 0.9 versus 3 ± 0.7; *p* = 0.920). There were no differences in ROM between cauterized and non-cauterized knees postoperatively (123° ± 10.8° versus 123° ± 10.2°; mean difference −0.4; 95% CI, −3.9 to 2.9; *p* = 0.783) at 2 years of follow-up. There were no differences in all parameters of the Knee Injury and Osteoarthritis Outcome Score between the two groups (*p* > 0.05).	No Benefit	Strengths: Clear intervention and control, multiple outcome measurements, each patient used as their own control. Weaknesses: Quasi-randomized design, small loss to follow-up, no significant differences in outcomes.	8/9
Goicoechea et al. (2020) [37]	Prospective, randomized, double-blind trial	Prospective study/level IV	169	At the 1-year follow-up, there were mild differences between the denervation and non-denervation groups in PPT value (494.4 kPa vs. 552.3 kPa, *p* = 0.047) and in VAS at stairs (2.9 vs. 1.5, *p* = 0.003) in favor of the non-denervation group. There was no difference in the improvement between groups in patellofemoral Feller score and KSS but slightly higher improvement in the non-denervation group in PPT (94.1 kPa vs. 160 kPa, *p* = 0.047), VAS walking (5.3 vs. 6.2, *p* = 0.041), and VAS at stairs (4.6 vs. 5.7, *p* = 0.022).	No Benefit	Strengths: Randomized, double-blinded, validated outcome measures, high follow-up rate, intention-to-treat analysis. Weaknesses: Lack of surgeon blinding, no significant improvement in anterior knee pain.	12/13
Pulavarti et al. (2014) [38]	Randomized, controlled trial	RCT/level I	126	Patient satisfaction was higher, with a greater number of patients rating the procedure as excellent in the denervation group (chi square 8.1, *p* < 0.05). Flexion at the latest follow-up was higher in the denervation group (*t*-test, *p* = 0.01). The anterior knee pain component within the patellar score and visual analogue scale (VAS) for anterior knee pain were significantly better in the denervation group at 3 months (*p* < 0.05) but not at 12 and 24 months.	Mixed Benefit	Strengths: Randomization, blinding of patients and assessors, validated outcome measures, 2-year follow-up. Weaknesses: Lack of surgeon blinding, minor losses to follow-up, short-term benefits not sustained.	11/13
Spencer et al. (2023) [11]	Randomized, controlled trial	RCT/level I	142	No difference in Oxford knee score was detected at 1 year (mean difference 1.87; 95% confidence interval −1.28 to 5.03). No difference was detected in Bartlett patella score (MD 0.490; 95% CI −1.61 to 2.59) or 12-Item Short-Form Survey (MD 0.196; 95% CI −2.54 to 2.93). A statistically significant difference in WOMAC was detected but at a level less than the minimal clinically significant difference for WOMAC (MD 4.79; 95% CI 1.05 to 8.52).	No Benefit	Strengths: Randomized, double-blinded, well-validated outcome measures, intention-to-treat analysis. Weaknesses: The surgeon was not blinded, there was an early imbalance in randomization, and there was some loss to follow-up.	11/13
Sun et al. (2024) [39]	Randomized, controlled trial	RCT/level I	120	UKA patients treated with PD achieved better Kujiala scores and FJS-12 scores, experiencing reduced anterior knee pain and improved kneeling ability postoperatively. The highest percentage of “good” actual kneeling ability was “90° kneeling on the cushion in the PD group (55.2%)” and the lowest percentage of “good” actual ability was “120° kneeling on the floor in the non-PD group (16.1%)”.	Benefit	Strengths: Proper randomization, allocation concealment, blinding of outcome assessors, well-validated outcome measures. Weaknesses: No participant or surgeon blinding, short follow-up period, simplified kneeling assessment.	11/13
Thiengwittayaporn et al. (2020) [40]	Randomized, controlled trial	RCT/level I	228	The incidence of AKP was significantly lower in the PD group (6.4% vs. 16.2%, *p* = 0.032). The intensity of AKP was considerably better in the PD group at 3 months (17.1 ± 8.0 vs. 54.0 ± 14, *p* = 0.017) but not at the later follow-up time points. Patient satisfaction scores were significantly better in the PD group at 3 months (14.7 ± 0.8 vs. 13.4 ± 1.0, *p* = 0.034) but not at the later time points.	Benefit	Strengths: Randomized, concealed allocation, blinding of assessors, validated outcome measures, high follow-up rate. Weaknesses: Lack of surgeon blinding, early benefits not sustained in the long term.	12/13
Van Jonbergen et al. (2014) [41]	Randomized, controlled trial	RCT/level I	202	The overall prevalence of anterior knee pain was 32% (95% CI 26 to 39), and 26% (95% CI 18 to 35) in the intervention group compared with 38% (95% CI 29 to 48) in the control group (chi-squared test; *p* = 0.06). The mean total Western Ontario and McMasters Universities Arthritis Index and the American Knee Society knee and function scores at 3.7 years’ follow-up were similar in the intervention and control groups (repeated measures analysis of variance *p* = 0.43, *p* = 0.09 and *p* = 0.59, respectively).	Mixed Benefit	Strengths: Randomization, patient and assessor blinding, validated outcome measures, transparent reporting of follow-up. Weaknesses: Lack of allocation concealment, surgeon blinding, high loss to follow-up.	10/13
